# Evaluation of the Antioxidant Activity of the Marine Pyrroloiminoquinone Makaluvamines

**DOI:** 10.3390/md14110197

**Published:** 2016-10-27

**Authors:** Eva Alonso, Rebeca Alvariño, Marta Leirós, Jioji N. Tabudravu, Klaus Feussner, Miriam A. Dam, Mostafa E. Rateb, Marcel Jaspars, Luis M. Botana

**Affiliations:** 1 Departamento de Farmacología, Facultad de Veterinaria, Universidad de Santiago de Compostela, Lugo 27002, Spain; eva.alonso@usc.es (E.A.); rebeca.alvarino@usc.es (R.A.); marta.leiros@usc.es (M.L.); 2 Marine Biodiscovery Centre, Department of Chemistry, University of Aberdeen, MestonWalk, Aberdeen AB24 3UE, UK; j.tabudravu@abdn.ac.uk (J.N.T.); miriam.dam@abdn.ac.uk (M.A.D.); Mostafa.rateb@uws.ac.uk (M.E.R.); m.jaspars@abdn.ac.uk (M.J.); 3 Institute of Applied Sciences, University of the South Pacific, P.O. Box 1168, Suva, Fiji; Feussner_k@usp.ac.fj

**Keywords:** makaluvamine, *Zyzzya*, oxidative stress, neurons, marine sponge, nrf2

## Abstract

Makaluvamines are pyrroloiminoquinones isolated from *Zyzzya* sponges. Until now, they have been described as topoisomerase II inhibitors with cytotoxic effects in diverse tumor cell lines. In the present work, seven makaluvamines were tested in several antioxidant assays in primary cortical neurons and neuroblastoma cells. Among the alkaloids studied, makaluvamine J was the most active in all the assays. This compound was able to reduce the mitochondrial damage elicited by the well-known stressor H_2_O_2_. The antioxidant properties of makaluvamine J are related to an improvement of the endogenous antioxidant defenses of glutathione and catalase. SHSY5Y assays proved that this compound acts as a Nrf2 activator leading to an improvement of antioxidant defenses. A low concentration of 10 nM is able to reduce the reactive oxygen species release and maintain a correct mitochondrial function. Based on these results, non-substituted nitrogen in the pyrrole plus the presence of a *p*-hydroxystyryl without a double bond seems to be the most active structure with a complete antioxidant effect in neuronal cells.

## 1. Introduction

Nowadays, the search for new and interesting compounds for drug development is focused on the marine environment as a source of new structures. Marine alkaloids isolated from sponges supply a wide range of new molecules to pharmaceutical libraries with different mechanisms of action [1]. These molecules have been mainly studied as anti-tumor or anti-infective agents and some of them, such as trabectedin or ziconotide, have successfully reached the pharmaceutical market [2]. One explanation for this wide range of different molecules is the fact that these organisms are usually sessile or have slow movements that restrict their own defense and therefore, they have to produce molecules to protect themselves from predators and/or bacteria overgrowth. Moreover, these molecules are often highly effective due to the marine environment where they are released [3]. The stressful conditions of the marine habitat such as ultraviolet radiation, desiccation, or metal exposure, lead to Reactive Oxygen Species (ROS) generation. Then, antioxidant agents are often isolated from marine organisms that produce them in order to counteract this oxidative stress condition [4,5]. Natural antioxidants can act exogenously, as direct scavengers, or endogenously to improve the cellular response to stress. Most of the marine antioxidants screened are crude extracts tested in solution-based chemical assays, but living cell assays with isolated compounds are essential for the advance to pharmacological testing [6]. 

In human cells, oxidative stress is involved in several pathologies such as cancer, ageing, or neurodegenerative diseases. Oxidative stress is defined as the imbalance between antioxidants and oxidants where the cellular defenses are not able to neutralize ROS overproduction. In physiological conditions, ROS are produced as a result of numerous reductions and reactions in the normal cellular operation; however, the organism has effective mechanisms to maintain a correct balance. In some situations, ROS production is increased and the cellular defenses are not able to control the equilibrium leading to oxidative damage in essential components such as lipids, proteins, and DNA, and finally to cell dysfunction and death. Among the antioxidant enzymes and proteins are superoxide dismutase, glutathione (GSH), and catalase that catalyze the processing of ROS to less toxic molecules. The enhancement of these antioxidant enzymes’ activity is a pursued strategy to decrease ROS levels, restore the balance, and protect the cells [7,8,9].

This unbalanced oxidative condition plays an important role in neurodegenerative diseases such as Alzheimer’s disease because the brain is the main consumer of oxygen and neurons are relatively sensitive to oxidative stress because of their poor antioxidant defenses [10], which makes neuronal cells a great model for antioxidant activity assays.

**Figure 1:**
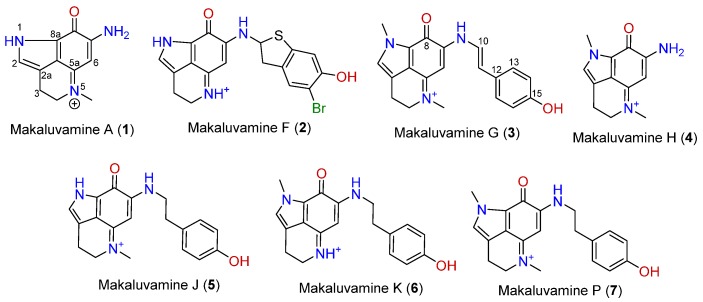
Chemical structures of makaluvamines.

Makaluvamines (Figure 1) are marine metabolites mainly isolated from *Zyzzya* genus sponges [11,12]. This group of compounds, along with epinardins, batzellines, or discorhabdins, share a pyrroloiminoquinone ring system. The makaluvamine family is made up of several compounds with a common 7-amino substituted pyrroloiminoquinone skeleton in which substitutions at N-1, N-5, and N-9 are usual [13]. These alkaloids have been widely reported as potent cytotoxic agents in several tumor cell lines through the inhibition of DNA processing enzymes, as topoisomerase II inhibition [14,15,16], however no clinical trials in humans have been performed so far. Although cytotoxicity and proapoptotic tests have been extensively published, little information has been released about the antioxidant potential of makaluvamines. To our knowledge, five makaluvamines were tested in a non-cell assay at high concentrations [17], but no living cell assays have been published. In the present work, the antioxidant potential of seven makaluvamines are tested in a mouse and a human neuronal model.

## 2. Results

**Figure 2:**
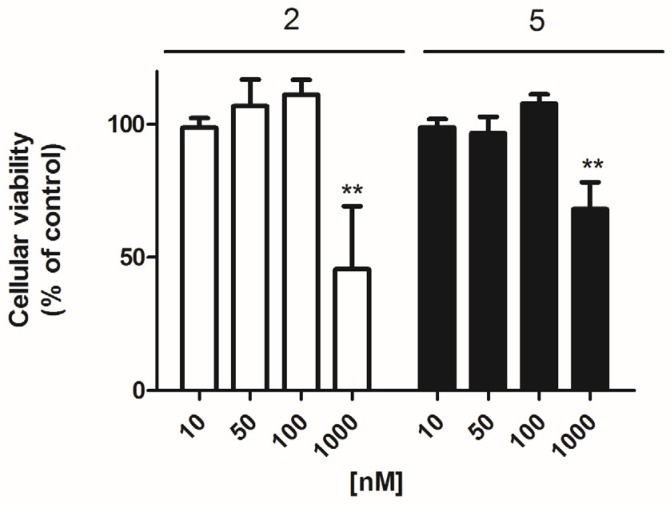
Effect of makaluvamines 2 and 5 on cellular viability. Cytotoxicity effect of compounds **2** and **5** after 24 h incubation measured by the 3-(4,5-Dimethylthiazol-2-yl)-2,5-Diphenyltetrazolium Bromide (MTT) test. ** *p* < 0.01.

Firstly, the effect of these compounds on the cellular viability of primary cortical neurons was checked with the 3-(4,5-Dimethylthiazol-2-yl)-2,5-Diphenyltetrazolium Bromide (MTT) test. For this, three concentrations (0.01, 0.1, and 1 μM) of each compound were added to the cellular medium and incubated for 24 h. Among the 7 makaluvamines tested, only compounds **2** and **5** elicited a viability decrease. Compound **2** at 1 μM was the most potent with a reduction of 54.5 ± 20.8% (*p* = 0.005) vs. control cells, whereas in the presence of compound **5** (1 μM) the viability diminished 31.8 ± 10.1% (*p*= 0.003) (Figure 2). In view of these results, compounds **2** and **5** were studied in all the assays at 0.05 and 0.1 μM, whereas all the other non-toxic compounds were tested at 0.1 and 1 μM.

**Figure 3:**
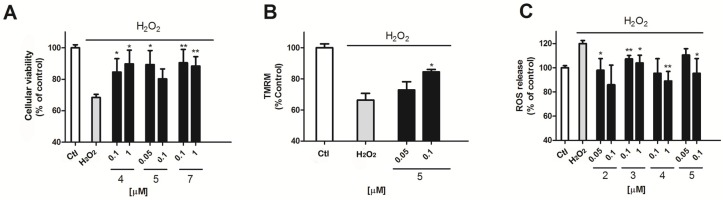
Effect of makaluvamines over H_2_O_2_ toxicity and ROS release (**A**) Neuroprotective effect of compound **4** and **5** in the presence of 200 μM H_2_O_2_ after 12 h incubation; (**B**) Effect of Compound **5** co-incubation over ΔΨm measured by tetramethylrhodamine (TMRM) assay; (**C**) Compounds **2**, **3**, **4**, and **5** at μM concentrations (0.05, 0.1 and 1 μM) inhibited ROS release versus H_2_O_2_ treated cells. The values are presented in percentage versus non-treated control cells, but the statistical comparison is made versus H_2_O_2_ treated cells.* *p* < 0.05 and ** *p* < 0.01. Data are mean ± SEM of three or more independent experiments performed intriplicate.

To evaluate the antioxidant potential of this compound family in this cellular model, H_2_O_2_ was used as an oxidative stress inductor. A 12 h cell incubation with 200 μM H_2_O_2_ produced cellular damage that resulted in a mitochondrial function decrease of 31.2 ± 2% (*p* = 0.005) versus non treated cells (Figure 3A) measured by the MTT test. The presence of compounds **4** or **5** was able to reduce this decrease by 15.5 ± 8.4% and 10.3 ± 8.7% (compound **4**, 0.1 and 1 μM, *p* = 0.03) or by 10.7 ± 9% (*p* = 0.01) (compound **5** at 0.05 μM). Interestingly, when the mitochondrial potential (ΔΨm) was evaluated by tetramethylrhodamine (TMRM) assay, only compound **5** showed some effect. Compound **5** at the highest concentration tested (0.1 μM) reduced the decrease of the ΔΨm observed in the presence of H_2_O_2_ (H_2_O_2_, 33.6 ± 2.5%, *p* = 0.0003 and compound **5**, 15.5 ± 1.5%, *p* = 0.012) (Figure 3B).

Mitochondria are the main producers of ROS in the cell. The damage of this organelle can result in defective respiratory chain work that results in an excessive O_2_ production [18,19]. So, we next studied if some of the makaluvamines were able to reduce the ROS levels elicited by 200 μM H_2_O_2_ in cortical neurons. As can be seen in Figure 3C, the oxidant produced an increase in ROS levels of 121.5 ± 2.5%, *p* = 0.0001 versus non treated cells. The presence of compounds **2**–**5** diminished this increase. Compound **2** was effective at the two concentrations tested, reducing ROS levels in H_2_O_2_ treated cells by 97.9 ± 9.6% and by 85.9 ± 16.3% at 0.05 and 0.1 μM, respectively (*p* = 0.03 and 0.04). Compound **3** decreased ROS release in the presence of H_2_O_2_ to a 107.2 ± 2.9% and to a 103.9 ± 6.4% (0.1 and 1 μM respectively, *p* = 0.008 and 0.03). Compound **4** showed a ROS quantification of 95.5 ± 5.3% (1 μM, *p* = 0.001) compared with the ~120% increase in the presence of the oxidant alone. Finally, compound **5** was only active at 0.1 μM with ROS levels of 95.3 ± 12.3% (*p* = 0.04) (Figure 3C). 

**Figure 4:**
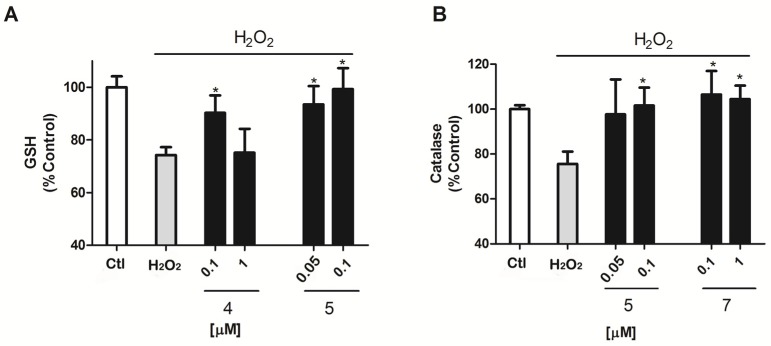
Evaluation of GSH and catalase activity after 12 h incubation with 200 μM H_2_O_2_. (**A**) Co-incubation of cells with compounds **4** and **5** at μM concentrations increase the levels measured by ThiolTracker Violet in cells co-treated with 200 μM H_2_O_2_ and compounds for 12 h; (**B**) Compound **5** increases catalase activity in the presence of 200 μM H_2_O_2_ measured by the Catalase Amplex Red kit (Invitrogen, Carlsbad, CA, USA). The values are presented in percentage versus non-treated control cells and compared to cells treated with 200 μM H_2_O_2_ alone.* *p* < 0.05. Data are mean ± SEM of three or more independent experiments performed in triplicate.

Antioxidants usually work as reactive species scavengers or as promoters or enhancers of the cellular antioxidant protection. GSH and Catalase are some of the main antioxidant defenses in the cell [20,21], therefore, the next step was to evaluate if makaluvamines were able to improve the operation of these two antioxidants in the murine model (Figure 4). In the presence of H_2_O_2_, GSH activity was reduced by 25.8 ± 3.1% (*p* = 0.0003) versus control cells. Compound **5** decreased this inhibition, with a GSH activity of near control levels, 93.5 ± 6.9% and 99.4 ± 7.9%, at 0.05 and 0.1 μM (*p* = 0.01 and 0.02), respectively. The presence of Compound **4** also had some effect on GSH activity, but only at 0.1 μM with GSH levels of 90.3 ± 6.6% (*p* = 0.04) (Figure 4A).

Catalase activity was affected by H_2_O_2_ treatment as well, as can be observed in Figure 4B; its activity was reduced by 24.4 ± 5.5% (*p* = 0.005) versus non treated cells. Compound **5** exhibited activity again, showing normal catalase activity levels; 101.6 ± 8% (*p* = 0.03) at the highest concentration 0.1 μM.

**Figure 5:**
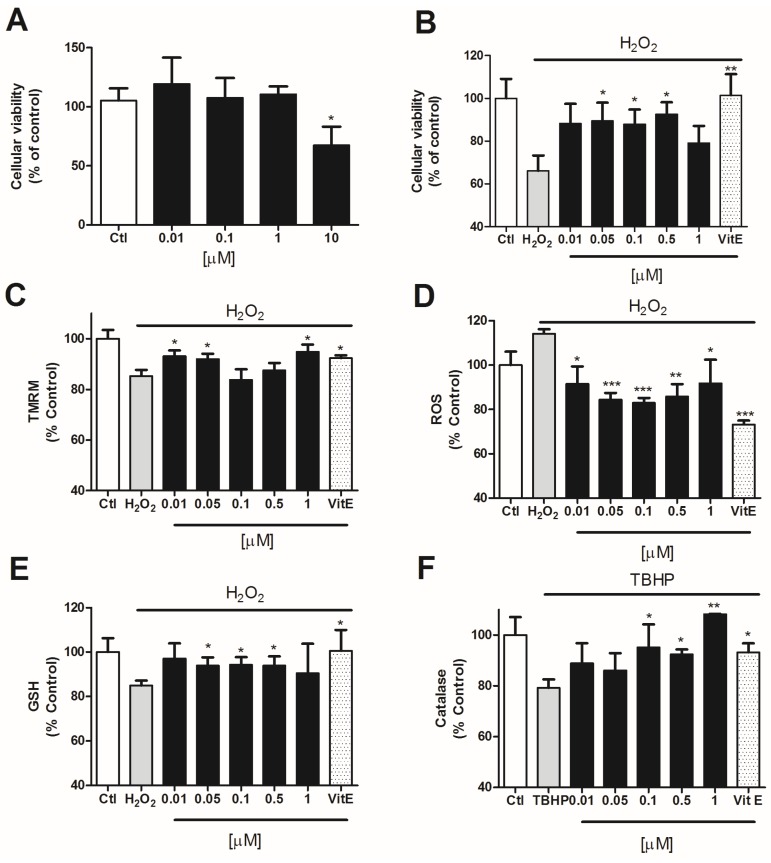
Makaluvamine J (5) effect over SHSY5Y cells pretreated with H_2_O_2_. Vitamin E was added as a positive control. (**A**) Cytotoxicity of compound **5** over neuroblastoma cells; (**B**) Neuroprotective effect of compound **5** in the presence of 150 μM H_2_O_2_ after 6 h incubation; (**C**) Effect of compound **5** co-incubation on ΔΨm measured by TMRM assay; (**D**) Compound **5** inhibition of ROS release versus H_2_O_2_ treated cells; (**E**,**F**) Evaluation of GSH and catalase activity after 6 h incubation with 150 μM H_2_O_2_. GSH levels measured by ThiolTracker Violet (**E**) and catalase activity measured by Catalase Amplex Red kit (Invitrogen) (**F**). The values are presented in percentage versus non-treated control cells and compared to cells treated with 150 μM H_2_O_2_ alone.* *p* < 0.05 and ** *p* < 0.01.

Since compound **5** was the most effective one, we decided to check its effect in a human neuronal model. For this we used the neuroblastoma cellular line SHSY5Y. First, we tested the cytotoxicity of compound **5** in SHSY5Y cells by MTT assay. Cells were incubated with the compound at several concentrations (0.01–10 μM) for 24 h. Only the highest concentration (10 μM) produced a decrease of 32.6 ± 11.2% in cell viability with respect to the control cells (Figure 5A). Therefore, we used lower concentrations (0.01, 0.05, 0.1, 0.5, and 1 μM) for the neuroprotection assays.

To determine the neuroprotective effect of the compound in SHSY5Y cells, we co-incubated the compound with H_2_O_2_ for 6 h [22,23]. In all the assays, Vitamin E was added as a positive control in order to compare the antioxidant activity of compound **5**. After this time, the mitochondrial function in cells treated with H_2_O_2_ alone was significantly reduced to 66.2 ± 7.1% (*p* = 0.0005) vs. control cells. The co-treatment with compound **5** at 0.05, 0.1, and 0.5 μM significantly protected SHSY5Y cells against oxidative damage, reaching percentages of 89.5 ± 8.5% (*p* = 0.04), 87.9 ± 6.7% (*p* = 0.03), and 92.6 ± 5.6% (*p* = 0.01), respectively (Figure 5B).

Compound **5** also displayed effects in the recovery of ΔΨ. Human neuroblastoma cells treated with H_2_O_2_ presented a decrease in ΔΨ of 16.0 ± 0.6% (*p* = 0.0001) with respect to untreated cells (Figure 5C). Compound **5** significantly improved the mitochondrial membrane depolarization produced by H_2_O_2_ at 0.01 (93.7 ± 2.9%; *p* = 0.02), 0.05 (93.9 ± 2.4%; *p* = 0.01) and 1 μM (95.7 ± 3.9%; *p* = 0.03).

The potential to diminish ROS levels in these cells was also evaluated. The incubation with H_2_O_2_ for 6 h increased ROS release in 14.1 ± 2.0% (*p* = 0.0004) vs. control cells (Figure 5D). The treatment with compound **5** significantly decreased the levels of ROS in neuroblastoma cells at all the concentrations tested, showing percentages of 91.5 ± 7.9% (*p* = 0.03), 84.4 ± 3.0% (*p* = 0.0001), 83.0 ± 2.2% (*p* = 0.00005), 85.8 ± 5.6% (*p* = 0.003), and 91.8 ± 10.7% (*p* = 0.03) at 0.01, 0.05, 0.1, 0.5, and 1 μM, respectively. 

Finally, we tested the ability of this compound to enhance the endogenous antioxidants GSH and catalase in SH-SY5Y human cells. GSH content was 15.1 ± 2.2% (*p* = 0.0002) lower in cells treated with 150 μM H_2_O_2_ than in control cells. As can be seen in Figure 5E, the incubation with compound **5** for 6 h at 0.05, 0.1, and 0.5 μM was able to significantly improve GSH levels in SH-SY5Y cells, reaching a 93.9 ± 3.6% (*p* = 0.04), 94.2 ± 3.4% (*p* = 0.03) and 93.9 ± 4.1% (*p* = 0.04), respectively.

In order to determine the effect of the compound on the activity of catalase, human neuroblastoma cells were incubated with 10 μM TBHP and compound **5** for 6 h. After this time, SH-SY5Y cells treated with TBHP alone presented a decrease in catalase activity of 20.9 ± 3.3% (*p* = 0.003) with respect to untreated cells (Figure 5F). When compound **5** at 0.1, 0.5, and 1 μM was added to the cells, the catalase activity was significantly increased reaching levels of 101.7 ± 10.9% (*p* = 0.04), 92.4 ± 1.9% (*p* = 0.02), and 108.2 ± 0.2% (*p* = 0.07).

Nuclear factor (erythroid-derived 2)-like 2 (Nrf2) is a transcription factor with a key role in cellular antioxidant defense. In physiological conditions this factor is bound to Kelch-like ECH associated protein 1 (KEAP1) in the cytoplasm, but under oxidative stress conditions it is released and translocated to the nucleus where it induces the expression of antioxidant genes such as catalase, superoxide dismutase, or glutathione peroxidase genes [24]. In view of the results obtained in the neuroprotection assays, the ability of compound **5** to produce the translocation of Nrf2 to the nucleus was analyzed.

**Figure 6:**
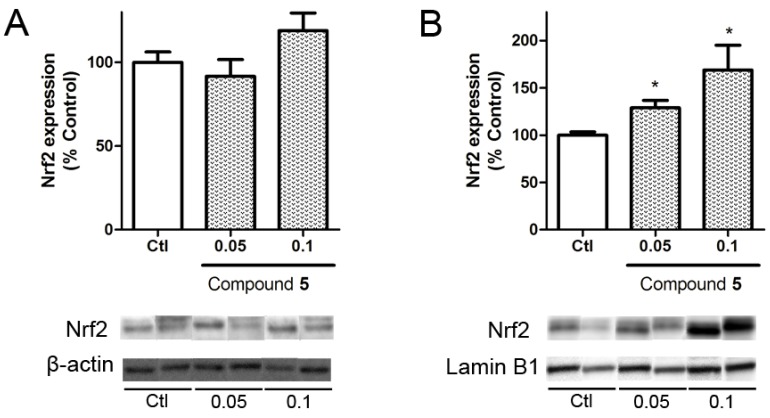
Induction of Nrf2 translocation by compound **5**. Nrf2 levels were measured in the lysates of SHSY5Y cells incubated with the compounds for 6 h. Results are presented separately in cytosolic (**A**) and nuclear (**B**) lysates. Nrf2 was normalized with Lamin B1 for nuclear samples and Actin for cytosolic lysates. Nuclear and cytosolic results obtained were statistically analyzed by Student’s *T*-test and compared to the controls of the nuclear and cytosolic samples, respectively. * (*p* < 0.05). Data are mean ± SEM of 3 or more independent experiments.

Cells were treated with compound **5** at 0.05 and 0.1 μM for 6 h and Nrf2 expression was determined by Western blot. The levels of the transcription factor in the cytosol showed no differences with respect to untreated cells, whereas nuclear levels were significantly increased by the compound presence at both concentrations (Figure 6). The treatment at 0.05 μM increase Nrf2 expression by 28.9 ± 7.8% (*p* = 0.02) vs. control cells. At 1 μM, makaluvamine J enhanced the translocation of the transcription factor to 168.8 ± 26.2% (*p* = 0.04).

## 3. Discussion

In this work we characterized the effect of seven marine alkaloids isolated from *Zyzyza* sponge in an in vitro oxidative stress model. We used the stressor H_2_O_2_ to induce the oxidative stress conditions in our neuronal models [25] and therefore, induced an imbalance between ROS generation and the antioxidant defenses. Although the antioxidant potential of makaluvamines has been previously partially studied, only makaluvamine C, E, G (3), H (4), and L were tested in a non-cellular model. In these assays, their antioxidant properties were studied by a scavenging assay and by linoleic acid autooxidation, and only makaluvamine E, G (3), and L showed a moderate activity but at higher concentrations than the highest concentration tested in the present work (100 μM versus 1 μM) [17].

Here, we analyzed several concentrations which selection was based on the cytotoxicity results. Only compounds **2** and **5** were toxic at 1 μM in the mice cortical neurons and therefore these two compounds were tested at 0.05 and 0.1 μM instead of 0.1 and 1 μM to guarantee that the observed effects were not related to necrotic or apoptotic events. Compound **1** was completely inactive, whereas compounds **6** and **7** showed little activity regarding cellular viability and catalase respectively. Compounds **2** and **3** were only able to reduce ROS levels, without any improvement in mitochondria function or in the antioxidant enzyme activity. This highlights that this isolated result can be related to a direct scavenger action and not with an endogenous effect, in agreement with the previous data in non-cellular systems of compound **3** [17].

Utkina et al. [17] showed that compound **4** was inactive in their scavenging assays whereas compound **3** and makaluvamine L elicited a moderate activity at 100 μM. Compounds **3** and **4** showed some activity in our antioxidant assays in primary cortical neurons, but neither of them provided full neuroprotection as compound **5** did. Compound **5** was, among the 7 makaluvamines tested, the only one that protected the mitochondria with the recovery of its potential and the reduction of ROS release. These effects were not a direct action over the oxidant species since the presence of this compound in the cellular medium showed an obvious improvement of the activity of catalase and GSH, two molecules that constituted an important cellular defense. Moreover, we tested if the effect of this compound was reproduced in the human neuroblastoma cell line SHSY5Y and we observed that after treatment this compound was also able to protect the cells from H_2_O_2_ damage and that this effect was related to Nrf2 factor translocation to the nucleus and endogenous antioxidant enzyme enhancement, proving the results in murine cells.

**Figure 7:**
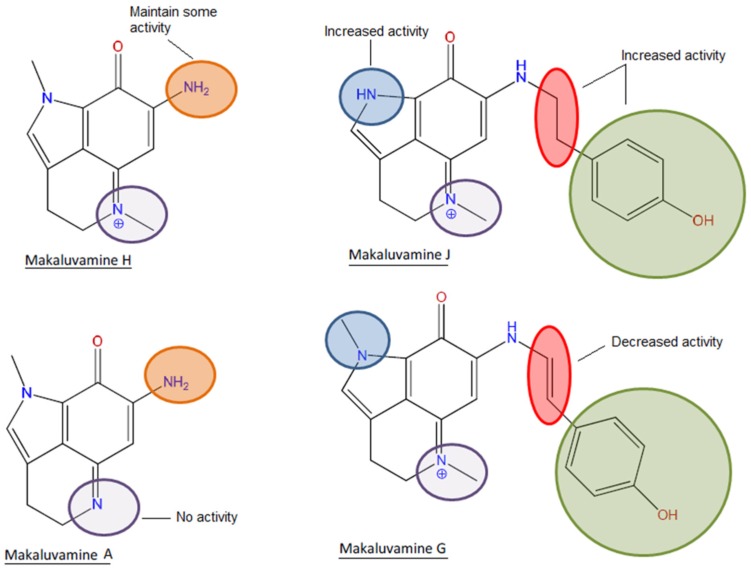
Structure-activity relationship of makaluvamines in antioxidant assays.

If we focus on the structure, it can be observed that the most active compound, compound **5**, and the poorly active compound **7** are almost identical. Both compounds share a p-hydroxyphenethyl unit with an aromatic ring and the only difference is the presence of a pyrrole with a non-substituted nitrogen in the case of compound **5** and a pyrrole with a *N*-methyl in the case of compound **7**. This non-substituted nitrogen seems to be essential for the activity since the compounds **1**, **3**, **6**, and **7** that shared the methyl substitution are all practically inactive or have little effect. An exception was observed in the case of compound **4**, one of the simplest structures, a pyrroloiminoquinone without the p-hydroxyphenethyl unit. Although this compound presented the methyl substitution, it possessed an amine instead of the hydroxyphenethyl which could be responsible for the observed activity. However, it is noteworthy that compound **1** did not exert any activity and in this case the only difference is that compound **4** possesses a positive charged nitrogen in position 5 (Figure 1 and Figure 7).

It was previously described in the ABTS assay that the presence of a p-hydroxystyryl unit instead of the p-hydroxyphenethyl was responsible for the scavenging activity of makaluvamine G (3) and L. In the present work, it is highlighted that although the presence of p-hydroxystyryl is not indispensable, the combination of this motif with the amine and the absence of a double bond produces the most complete antioxidant activity. The bioactivity of an antioxidant is usually determined by its hydrogen or electron donating properties, the ability to chelate transition metal ions, or its ability to scavenge lipid peroxyl radicals. The presence of hydroxyl groups attached to aromatic ring substructures contributes to antioxidant effects through hydrogen donation. Moreover, the presence of oxo groups, such as the ketonic C=O at position 8 in the makaluvamines, and the absence of the C10/C11 double bond contributed to improving the antioxidant characteristics [26,27].

In physiological conditions, mitochondrial respiration produces ROS, but they are scavenged by the antioxidant defenses. However, in some pathological states such as stroke, Alzheimer’s disease, and Parkinson’s disease, these defenses fail and ROS accumulation is followed by a cascade of mitochondrial failures that ends in cellular death. Antioxidants that work through the endogenous pathway are usually more interesting than the direct scavengers in this kind of pathologies. The antioxidant responsive element (ARE) is one of the most important cellular defenses regulated by Nrf2. Therefore, the Nrf2 pathway has been extensively studied and its role in neurodegenerative diseases has been confirmed. The protective effect of Nrf2 activation has led to the development of a FDA approved drug, Tecfidera (dimethyl fumarate), Biogen Idec (Cambridge, MA, USA), for the treatment of patients with relapsing multiple sclerosis and the application of Nrf2 activators for other diseases is currently being researched [28,29,30]. The development of new antioxidant molecules that have effects on mitochondria and on the antioxidant enzymes can contribute to the expansion of our knowledge about the potential of antioxidant therapies in neurodegenerative diseases. Moreover, the identification of the essential structural components for this bioactivity opens new pathways for the synthesis of new and more potent analogues.

## 4. Materials and Methods

### 4.1. Compound Information

Makaluvamine A(1), F(2), G(3), H(4), J(5), K(6), and P(7) were isolated from a marine sponge (*Zyzzya* sp.) collected near Bouma village, Taveuni Island, Cakaudrove Province, Fiji islands. Isolations and purifications were performed by HPLC and chemical dereplications were performed by LCMS and NMR methods. Chemical information and the structures of the compounds are presented in Figure 1 while LCMS was obtained using a Thermo Instruments MS system (LTQ XL/LTQ Orbitrap Discovery) coupled to a Thermo Instruments HPLC system (Accela PDA detector, Accela PDA autosampler, and Accela pump) and using a capillary voltage of 45 V, capillary temperature of 260 °C, auxiliary gas flow rate of 10–20 arbitrary units, sheath gas flow rate of 40–50 arbitrary units, spray voltage of 4.5 kV, and a mass range of 100–2000 amu (maximum resolution 30,000). 1H NMR (obtained using Varian 400 MHz NMRsystem) data of all of the compounds are found in the Supplementary Materials.

### 4.2. Cell Culture

Swiss mice were used to obtain primary cultures of cortical neurons. All protocols described in this work were revised and authorized by the University of Santiago de Compostela Institutional Animal Care and Use Committee and complied with European legislation on the use and management of experimental animals.

Primary cortical neurons were obtained from 15 to 18 day old mice fetuses as described previously [31]. Briefly, the cerebral cortex was removed and neuronal cells were dissociated by trypsinization at 37 °C, followed by mechanical titration in DNase solution (0.005% *w*/*v*) with a soybean trypsin inhibitor (0.05% *w*/*v*). Cells were suspended in DMEM supplemented with p-amino benzoate, insulin, penicillin, and 10% fetal serum. The cell suspension was seeded in 96 multiwell plates and incubated in a humidified 5% CO_2_/95% air atmosphere at 37 °C. Cytosine arabinoside 20 μM, was added before the 48 h of culturing to prevent growth of non-neuronal cells. In all the experiments, cortical neurons were treated for 4–5 days in vitro (div).

The human neuroblastoma SHSY5Y cell line was purchased from American Type Culture Collection (ATCC), number CRL2266. The cells were maintained in Dulbecco´s Modified Eagle´s medium: Nutrient Mix F-12 (DMEM/F-12) supplemented with 10% fetal bovine serum, glutamax, 100 U/mL penicillin, and 100 μg/mL streptomycin at 37 °C in a humidified atmosphere of 5% CO_2_ and 95% air. Cells were dissociated weekly using 0.05% trypsin/EDTA. All reagents were provided by Thermo Fisher Scientific (Waltham, MA, USA).

### 4.3. Chemicals and Solutions

Plastic tissue-culture dishes were purchased from Falcon (Madrid, Spain). Fetal serum and Dulbecco’s Modified Eagle’s medium (DMEM) were purchased from Thermo Fisher Scientific (Waltham, MA, USA). All other chemicals were reagent grade and purchased from Sigma-Aldrich (Madrid, Spain).

### 4.4. Cytotoxicity Assay

Cell viability was assessed by the MTT (3-[4,5-dimethylthiazol-2-yl]-2,5-diphenyltetrazoliumbromide) test, as previously described [32,33]. The assay was performed in cultures grown in 96 well plates and exposed to different compound concentrations (0.01, 0.05, 0.1 and 1 μM) added to the culture medium. For all the 96 well plate assays, 50,000 cells/well were seeded. Cultures were maintained in the presence of the compounds at 37 °C in humidified 5% CO_2_/95% air atmosphere for 48 h. Saponin was used as the cellular death control and its absorbance was subtracted from the other data. After treatment time, cells were rinsed and incubated for 1 h with a solution of MTT (500 μg/mL) dissolved in saline buffer. After washing off excess MTT, cells were disaggregated with 5% sodium dodecyl sulfate and the absorbance of the colored formazan salt was measured at 595 nm in a spectrophotometer plate reader.

### 4.5. Neuroprotection Assays

All assays were performed in 96-well plates (50,000 cells/well), as co-incubations of 200 μM H_2_O_2_ and the compound in two different concentrations for 12 h on 4–5 div murine cortical neurons [34]. Neuroblastoma cells were cultured instead with 150 μM for 6 h [22,23].

#### Neuroprotection and Mitochondrial Membrane Potential (ΔΨm) Assays

The neuroprotective effects on thecellular viability of Makaluvamines in the presence of the oxidant H_2_O_2_ were measured by the MTT test following the method described above and changes in ΔΨm were studied with the tetramethylrhodamine methyl ester (TMRM) assay. For TMRM assays, cells were washed twice with saline solution and incubated with 1 μM TMRM for 30 min. Then neurons were solubilized with 50% DMSO/water. Fluorescence values were obtained using a spectrophotometer plate reader (535 nm excitation, 590 nm emission).

### 4.6. Determination of ROS Production

Intracellular ROS levels were determined with carboxy-H_2_DCFDA (5-(and-6)-carboxy-2′,7′-dichlorodihydrofluorescein diacetate). After treatment with Makaluvamines and H_2_O_2_, cells were washed twice with serum-free medium. Then, 20 μM of carboxy-H_2_DCFDA dissolved in serum-free medium was added to the cells. After 1 h at 37 °C, the medium containing the fluorescence dye was replaced with PBS. The plate was incubated for 30 min at 37 °C and fluorescence was read at 527 nm, with an excitation wavelength of 495 nm.

### 4.7. Glutathione Assay

Reduced glutathione is the majority of intracellular free thiols in cells, so we used ThiolTracker™ Violet dye to estimating their levels in our treated cells. Neurons were washed with phosphate buffer solution and loaded with 10 μM ThiolTracker™ Violet dye for 1 h at 37 °C. After incubation, neurons were washed once and fluorescence was read at 404 nm excitation and emission at 526 nm.

### 4.8. Catalase Activity Assay

Catalase activity was measured with an Amplex^®^ Red Catalase Assay Kit after exposure of the samples to H_2_O_2_ following the commercial protocol. Fluorescence was read at 530 nm excitation and 590 nm emission. Enzymatic activity was calculated by subtracting sample values to the no-catalase control.

SHSY5Y human neuroblastoma cells were co-incubated with 10 μM tert-butyl hydroperoxide (TBHP) instead of H_2_O_2_ to obtain a clearer signal and makaluvamine J (0.01, 0.05, 0.1, 0.5, and 1 μM) for 6 h.

### 4.9. Western Blot Analysis

SH-SY5Y cells were seeded in 6-well plates at a density of 1 × 10^6^ cells per well and treated with makaluvamine J (0.05 and 0.1 μM) for 6 h. After treatment with the compound, neuroblastoma cells were rinsed twice with ice-cold PBS. Next, 100 μL of an ice-cold hypotonic solution buffer (20 mM Tris-HCl pH 7.4, 10 mM NaCl, and 3 mM MgCl_2_, containing a complete phosphatase/protease inhibitor cocktail from Roche) were added. Cells were scrapped, incubated on ice for 15 min and finally centrifuged at 3000 rpm, 4 °C for10 min. The supernatant was collected as the cytosolic fraction and the pellet was resuspended in an ice-cold nuclear extraction buffer (100 mM Tris pH 7.4, 2 mM Na_3_VO_4_, 100 mM NaCl, 1% Triton X-100, 1 mM EDTA, 10% glycerol, 1 mM EGTA, 0.1% SDS, 1 mM NaF, 0.5% deoxycholate, and 20 mM Na_4_P_2_O_7_, containing 1 mM PMSF and a protease inhibitor cocktail). Samples were incubated for 30 min, vortexedin 10 min intervals, and centrifuged at 14,000 *g* and 4 °C for 30 min. The supernatant was saved for nuclear protein fraction. This fraction was quantified by the Bradford method, whereas the cytosolic protein fraction was quantified using the Direct Detect system (MerckMillipore, Darmstadt, Germany). Samples containing 20 μg (cytosolic fraction) or 10 μg (nuclear fraction) were used for electrophoresis, which was resolved in a 10% sodium dodecyl sulphate polyacrylamide gel (Biorad, Hercules, CA, USA) and transferred onto PVDF membranes (Millipore). The Snap i.d. protein detection system was used for membrane blocking and antibody incubation. Anti-NF-E2 related factor 2 antibody was used to detect Nrf2 (1:1000, Millipore), and the signal was normalized using β-actin (1:10,000, Millipore) for the cytosolic fraction and laminB1 (1:1000, ABCAM) for the nuclear fraction. Protein bands were detected using Supersignal West Pico Luminiscent Substrate, Supersignal West Femto Maximum Sensitivity Substrate (Thermo Fisher Scientific), the Diversity GeneSnap system (Syngene, Cambridge, UK), and software.

### 4.10. Statistical Analysis

All the results are expressed as means ± SEM of three or more experiments (each performed in triplicate). Statistical comparison was done by Student’s *T*-test *p* values <0.05 were considered statistically significant.

## Supplementary Materials

The following are available online at www.mdpi.com/1660-3397/14/11/197/s1, Figure S1: MS analysis of the isolated makaluvamines, Figure S2: 1H NMR spectra of the isolated makaluvamines.
