# Supplementary material for: Evaluation of the Antioxidant Activity of the Marine Pyrroloiminoquinone Makaluvamines
Source: Mar Drugs. 2016 Oct 27;14(11):197. doi: 10.3390/md14110197 (PMC5128740; doi:10.3390/md14110197)
Supplement: Supplementary file 1 [file marinedrugs-14-00197-s001.pdf]

Supplementary Materials: Evaluation of the Antioxidant Activity of the Marine Pyrroloiminoquinone Makaluvamines

Eva Alonso, Rebeca Alvariño, Marta Leirós, Jioji N. Tabudravu, Klaus Feussner, Miriam A. Dam, Mostafa E. Rateb, Marcel Jaspars and Luis M. Botana

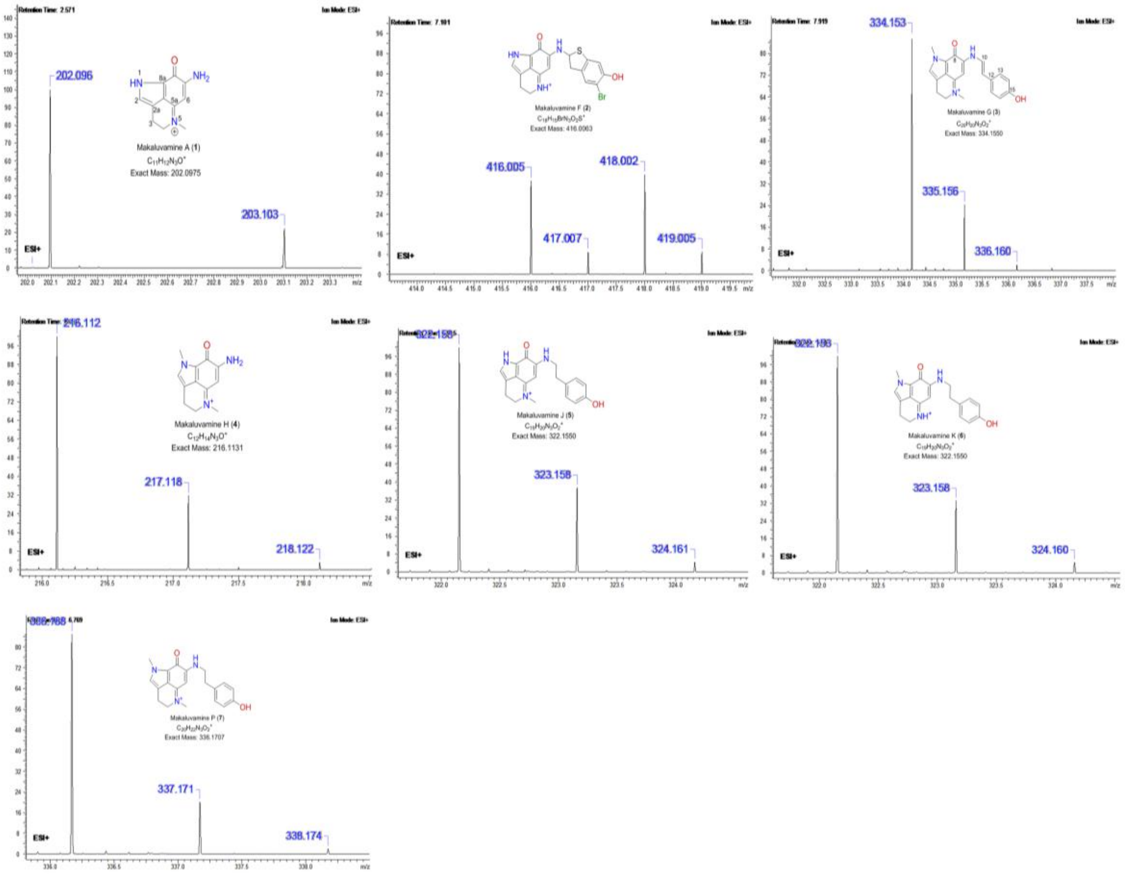

Figure S1. MS analysis of the isolated makaluvamines.

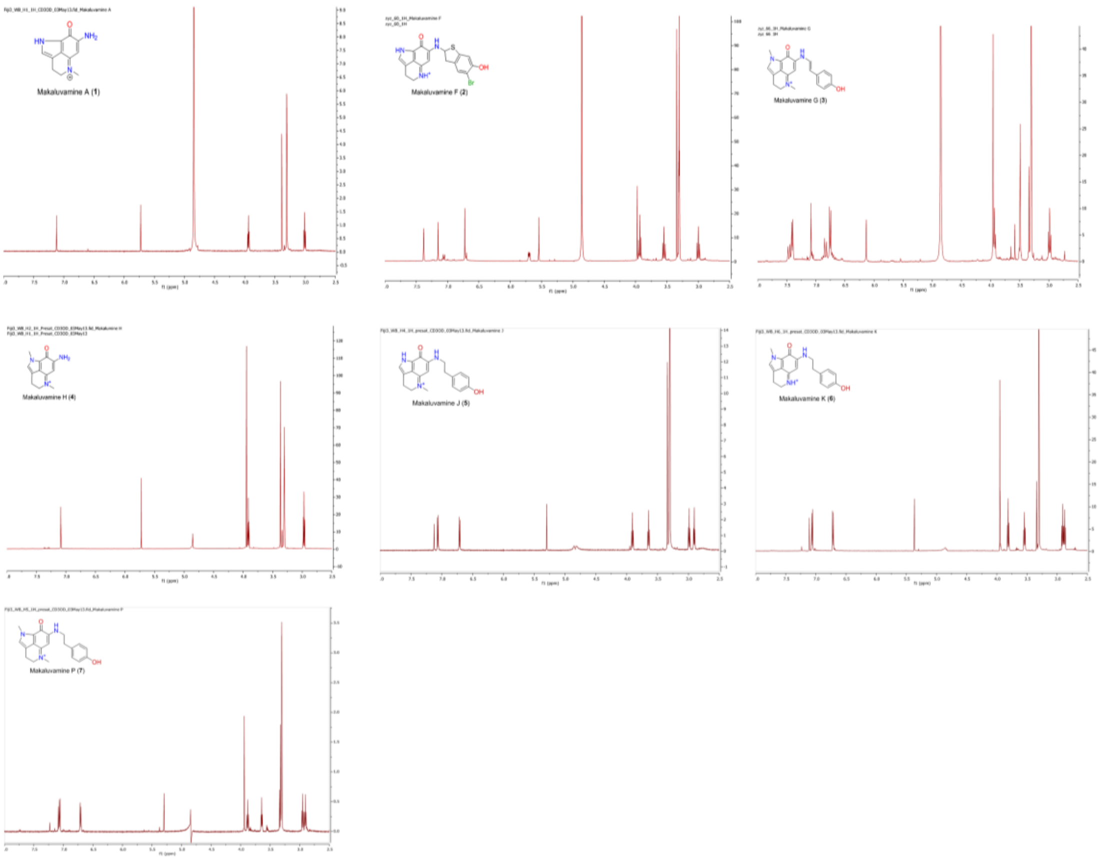

Figure S2. ^{1}H NMR spectra of the isolated makaluvamines.
